# Evidence for a general performance‐monitoring system in the human brain

**DOI:** 10.1002/hbm.24273

**Published:** 2018-07-04

**Authors:** Ivan Zubarev, Lauri Parkkonen

**Affiliations:** ^1^ Department of Neuroscience and Biomedical Engineering Aalto University Espoo Finland; ^2^ Aalto Neuroimaging Aalto University Espoo Finland

**Keywords:** brain–computer interface, electroencephalography, error processing, error‐related negativity, feedback‐related negativity, machine learning, magnetoencephalography, performance monitoring, reward processing

## Abstract

Adaptive behavior relies on the ability of the brain to form predictions and monitor action outcomes. In the human brain, the same system is thought to monitor action outcomes regardless of whether the information originates from internal (e.g., proprioceptive) and external (e.g., visual) sensory channels. Neural signatures of processing motor errors and action outcomes communicated by external feedback have been studied extensively; however, the existence of such a general action‐monitoring system has not been tested directly. Here, we use concurrent EEG‐MEG measurements and a probabilistic learning task to demonstrate that event‐related responses measured by electroencephalography and magnetoencephalography display spatiotemporal patterns that allow an effective transfer of a multivariate statistical model discriminating the outcomes across the following conditions: (a) erroneous versus correct motor output, (b) negative versus positive feedback, (c) high‐ versus low‐surprise negative feedback, and (d) erroneous versus correct brain–computer‐interface output. We further show that these patterns originate from highly‐overlapping neural sources in the medial frontal and the medial parietal cortices. We conclude that information about action outcomes arriving from internal or external sensory channels converges to the same neural system in the human brain, that matches this information to the internal predictions.

## 1 INTRODUCTION

Neural responses to negative or unexpected action outcomes have been the main target of research seeking to understand neural mechanisms of adaptive behavior in humans (Luft, 2014; Walsh & Anderson, 2012; Weinberg, Dieterich, & Riesel, 2014). Electroencephalographic (EEG) and magnetoencephalographic (MEG) studies have identified evoked responses elicited by errors in motor tasks (error‐related negativity, ERN, Ne) (Falkenstein, Hohnsbein, & Hoormann, 1995; Holroyd & Coles, 2002; Keil, Weisz, Paul‐Jordanov, & Wienbruch, 2010) as well as by external feedback (Feedback‐Related Negativity, FRN) (Doñamayor, Marco‐Pallarés, Heldmann, Schoenfeld, & Münte, 2011; Doñamayor, Schoenfeld, & Münte, 2012b; Gehring & Willoughby, 2002; Miltner, Braun, & Coles, 1997). These neural signals appear consistently across different tasks (Meyer, Riesel, & Hajcak Proudfit, 2013; Olvet & Hajcak, 2009), are indicative of post‐error behavioral adjustments (Holroyd & Coles, 2002; Nieuwenhuis et al., 2002), and are known to be altered in a number of neuropsychiatric conditions (Gründler, Cavanagh, Figueroa, Frank, & Allen, 2009; Gu, Huang, & Luo, 2010; Morris, Heerey, Gold, & Holroyd, 2008; Morris, Holroyd, Mann‐Wrobel, & Gold, 2011; Moser, Moran, Schroder, Donnellan, & Yeung, 2013; Proudfit, 2015; Weinberg, Klein, & Hajcak, 2012).

A prominent theory by Holroyd and Coles (2002) suggests that the motor‐response‐locked ERN and feedback‐locked FRN represent phasic changes in dopaminergic signaling to prefrontal cortex. Importantly, these authors suggest that outcome information from internal and external sources converges to a general performance‐monitoring system giving rise to both event‐related potentials (ERPs). In the case of ERN, the outcome is communicated either by an efferent copy of the motor program (Falkenstein et al., 1995; Stahl & Gibbons, 2007) or by proprioceptive input (Holroyd & Coles, 2002), while FRN is triggered whenever the outcome information arrives via external sensory (visual, auditory, etc.) inputs (Gehring & Willoughby, 2002; Miltner et al., 1997). However, this hypothesis has received little experimental support so far.

Here, to probe the functional similarity of ERN and FRN, we used the recently introduced across‐condition generalization technique (Kaplan, Man, & Greening, 2015; King & Dehaene, 2014). Specifically, we tested whether a classifier trained to discriminate negative versus positive outcomes in one condition (e.g., erroneous vs. correct motor output) can be successfully transfered to another classification problem (e.g., discriminating negative vs. positive feedback).

**Figure 1:**
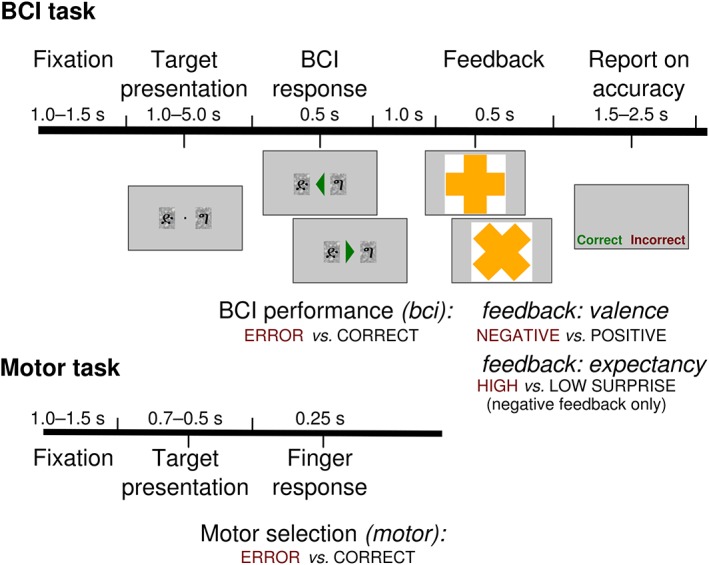
Design of the experiment. Condition names are shown in italics. *BCI task*: Participant selects a target by maintaining visual attention on it. BCI decodes and reports subject's selection correctly or incorrectly. Feedback is generated according to the probability associated with the target selected by a BCI. *Motor task*: Participants select targets by button presses based on the preferences learned during the BCI task. No feedback is presented [Color figure can be viewed at http://wileyonlinelibrary.com]

We collected the *feedback*‐related brain responses from a probabilistic learning task controlled by a brain–computer interface (BCI). Using a BCI ensured active involvement of the participants (as compared to the passive viewing tasks used previously) and, at the same time, minimized the possibility of the spurious generalization with *motor* trials due to the presence of highly‐similar movement‐related activity (Figure 1). We separately recorded motor‐related error responses during a speeded motor task in the same participants. We then applied across‐condition generalization technique (Kaplan et al., 2015; King & Dehaene, 2014) to identify patterns in EEG–MEG signals that (a) allow discriminating between outcomes within a condition (e.g., negative vs. positive feedback), and (b) transfer to a different classification problem (e.g., motor error vs. negative feedback). Importantly, this “generalization test” is performed at each time instant allowing identification of such patterns even if they occur at different latencies with respect to their triggering events. Similarly, we tested whether brain responses to loss of BCI control involve the same sources as responses to motor errors and probabilistic feedback.

Previous research confirmed that both ERN and FRN, computed as the difference between the ERPs to negative versus positive outcomes, display highly similar EEG voltage distributions characterized by a frontal–central negative focus, sometimes a more anterior distribution for FRN (Martin & Potts, 2011; Miltner et al., 1997; Potts, Martin, Kamp, & Donchin, 2011). EEG and MEG source modeling also supported common neural generator in the dorsal anterior cingulate cortex (dACC, Keil et al., 2010; Miltner et al., 2003; Nieuwenhuis, Slagter, von Geusau, Heslenfeld, & Holroyd, 2005). Other studies suggest a more posterior medial sources of FRN or ERN (posterior cingulate cortex and precuneus) (Agam et al., 2011; Doñamayor, Heilbronner, & Münte, 2012a). An fMRI study showed overlapping BOLD‐signal increase in dACC both when committing an erroneous motor response and when receiving negative feedback (Holroyd et al., 2004).

However, several lines of conflicting evidence exist. Patient studies demonstrate that ERN and FRN may be affected differently in a number of neuropsychiatric conditions including obsessive–compulsive disorder (Gründler et al., 2009), trait anxiety (Gu et al., 2010), major depression (Proudfit, Bress, Foti, Kujawa, & Klein, 2015; Weinberg et al., 2012) and schizophrenia (Morris et al., 2011). These discrepancies led researchers to suggest that, despite the apparent similarities, distinct neuronal populations may be involved in producing motor‐ and feedback‐related error responses (Müller, Möller, Rodriguez‐Fornells, & Münte, 2005).

One possibility to reconcile this conflicting evidence follows from the suggestion that FRN may comprise two distinct components (Heydari & Holroyd, 2016; Holroyd, Pakzad‐Vaezi, & Krigolson, 2008); the expectancy component (N200 response) is thought to track surprising or unexpected task‐related information (Talmi, Atkinson, & El‐Deredy, 2013) while the valence component (reward positivity) is considered to index processing of reward information (Holroyd et al., 2008). In line with this model, one study reported two distinct spatiotemporal principal components contributing to the FRN. The authors suggested that one of them may represent the same neural activity as the ERN while the other could be specific to external feedback (Potts et al., 2011). Yet, whether these motor‐ and feedback‐related evoked responses track expectation violation, processing of reward information, or both, remains largely unknown. The across‐condition generalization method allowed us to probe how the processing of valence and expectancy of the feedback contributes to the observed similarities.

## 2 MATERIALS AND METHODS

### 2.1 Participants

Fourteen healthy volunteers (6 females, 8 males, mean age 25 years, range 21–33) naïve to BCIs participated in the experiment. Aalto University Committee on Research Ethics approved the study. All subjects read the description of the experiment and signed the informed consent form before the experiment. The data of one subject were removed from the analysis involving the *motor* condition due to the very low number of errors in a motor task.

### 2.2 Experimental procedure

We used a probabilistic learning task (Frank, Seeberger, & O'reilly, 2004), which we adapted for a BCI. The subjects were exposed to four initially unfamiliar target stimuli, each of which had a specific, fixed probability of generating positive feedback (80%, 66%, 33%, and 20%, we used two different stimuli–value associations randomly assigned across subjects). On each trial, the subjects had to choose by a BCI from two alternative targets presented on the screen; the subjects’ task was to maximize positive feedback across the experiment. Based on the feedback, the subjects gradually learned to choose the more “valuable” target of each presented pair.

After the BCI task, the subjects were presented with the same target stimulus pairs, but they were requested to indicate their choice of the more valuable one by lifting the left or right index fingers instead of using the BCI. No feedback was presented in the motor task. Both the BCI and motor tasks are presented in detail in the following text.

### 2.3 BCI control

We used a steady‐state visual‐evoked‐response (SSVER) ‐based BCI paradigm to control the task. On each trial, two target stimuli (letters from the Tigrinya alphabet, unknown to the subjects) appeared on the screen. Each stimulus was masked by a white noise pattern modulated at 12 or 15 Hz. The noise mask was sampled from a pre‐generated set at this frequency and the opacity of the noise image was scaled with a sinusoid of the same frequency so that it varied from 10% to 90%. (Figure 1). The stimuli were designed so that the distance from the fixation point to the middle of the stimulus did not exceed 2 angular degrees. The data from the MEG acquisition system were continuously transmitted to the on‐line analysis computer in consecutive 500‐ms segments via a real‐time buffer mechanism (Oostenveld, Fries, Maris, & Schoffelen, 2011; Sudre et al., 2011). The time courses of four characteristic spatial patterns for the pre‐defined frequency components were extracted from a subset of 96 occipital and parietal gradiometers using the spatio–spectral decomposition (SSD) algorithm (Haufe, Dähne, & Nikulin, 2014). Following the procedure in an earlier study (Parkkonen, Andersson, Hämäläinen, & Hari, 2008), the SSD‐reduced data were fit with a general linear model comprising regressors for both target modulation frequencies (12 and 15 Hz), alpha rhythm (10 Hz), line‐frequency interference (50 Hz), DC offset, and a linear trend to obtain the amplitude estimates of the 12‐ and 15‐Hz signals. For each of the modulation frequencies, the model comprised separate sine and cosine predictors to accommodate for the unknown phase of the signal. The final amplitude estimates comprised the norms of the estimates, including the respective sine and cosine components. The resulting amplitude estimates were passed on to a linear support vector machine (SVM) classifier with the regularization parameter *C* = 0.5 (Vapnik, 2000). We defined that a decision was reached when two consecutive time segments were classified to the same class with *p* > .75 to control the misclassification rate at (1–0.75)^2^ = 0.0625. The probability model for SVM was created using cross validation as implemented in the Scikit‐Learn package (Pedregosa et al., 2011).

To choose the target, the subjects were instructed to focus and maintain visual attention on it until the system indicated the response. When the system reached a decision about the subject's choice, a green arrow pointing to the selected target appeared in the middle of the screen for 500 ms indicating a choice. If the classifier failed to arrive to a decision in 5 s, the trial ended with the message “Too late” appearing on the screen.

On making the selection, the subject received feedback whether he or she had earned (“+”; the plus sign) or had not earned (“x”; the cross sign) the score in this trial. The feedback was displayed 1,000 ms after the target selection. At the end of each trial, the subject was asked to report whether the system had indicated his or her choice correctly by lifting the left or right index finger. The association of the left/right finger and the correct “incorrect” indication was randomized across trials to ensure that no systematic preparatory activity contaminated the data following the correct or incorrect BCI performance. The subjects were instructed to indicate whether a system indicated their choice correctly (BCI‐correct) or incorrectly (BCI‐error) as accurately as possible; no time limit was set for reporting on BCI performance.

We motivated the subjects to earn a high score in the learning task by telling them that the subject attaining the highest score will be awarded a prize. We also encouraged them to report the BCI performance as accurately as possible by saying that an incorrectly labeled trial may worsen the BCI performance and by mentioning that every trial counted as “incorrect” will be discarded and will thus prolong the experiment. Therefore, dishonest reporting was against the subject's interest.

### 2.4 BCI calibration

The BCI calibration consisted of two blocks (4 min per block) comprising 16 trials each. The design of the calibration trials was similar to those in the actual experiment. On each trial, the target stimulus (star shape) and a blank rectangle appeared on the screen. Participants were instructed to sustain visual attention on the target stimulus for 5 s. The data from the first calibration block were used to initialize the spatial filters and the classifier. The performance of the system was then tested online during the second calibration block.

Similarly to the rest of the experiment, subjects received positive feedback whenever the target stimulus was chosen and negative feedback if the non‐target, that is, the blank rectangle, was chosen.

### 2.5 BCI task

The BCI task consisted of 360 trials and was split into five blocks consisting of 72 trials each. On each trial, a random pair from the set of four stimuli appeared on the screen. As the stimuli, we used letters from the Tigrinya alphabet, which was unfamiliar to all of our subjects. The probabilities of generating positive feedback were 0.8, 0.66, 0.33, and 0.2.

Trials containing evoked responses related to processing *positive* versus *negative feedback, high‐* versus *low‐surprise feedback*, and *erroneous* versus *correct* classification of subjects’ intentions by the *BCI* were taken from the BCI task.

To ensure a sufficient number of BCI errors for the analysis, we introduced a randomly‐appearing error to the BCI control affecting 15% of the trials. These artificial BCI errors produced a distinct trigger code such that these trials could be separated from all other trials. BCI‐error trials were then defined as those with this trigger code followed by a subjective report of incorrect BCI performance. More details on the BCI control accuracy and trial counts are presented in Supporting Information Tables I–II. If the subject's intention was misclassified by the BCI, the feedback was always generated according the stimulus that was (incorrectly) indicated as chosen by the BCI.

For the analysis of the feedback‐related responses (*positive* vs. *negative feedback, high‐* vs. *low‐ surprise feedback*), we retained only those trials where the subject's intention was correctly decoded by the BCI. For detailed definition of *positive* versus *negative feedback, high‐* versus *low‐ surprise feedback*, see subsections 2.10 and 2.11.

### 2.6 Motor task

The *motor* task block consisted of 288 speeded‐response trials, during which the subjects responded by lifting the left or right index finger. In the beginning of the block, the subjects had to respond within 700 ms. After each set of 50 trials, the maximal allowed response time decreased by 51 ms. The subjects were instructed to avoid exceeding the time limit even if that might result in incorrect responses (i.e., incorrect responses were preferred over missed trials). As the stimuli and their respective values were already familiar to the subjects (see Section 3.1), the subjects received no feedback in this block. We defined all trials were the subjects chose the least valuable alternative as (*motor*) *error* trials.

### 2.7 EEG–MEG Acquisition

MEG data were acquired using a 306‐channel Elekta Neuromag System (Elekta Oy, Helsinki, Finland) comprising 102 magnetometers and 204 planar gradiometers. Data were sampled at 1,000 Hz after filtering to 0.1–330 Hz. EEG was recorded concurrently using a 64‐electrode Waveguard™ (Advanced NeuroTechnology, Enschede, The Netherlands) MEG‐compatible cap with the reference electrode at the AFz position. Prior to analysis, the EEG signals were re‐referenced to their average value.

To control for eye‐movement‐related artifacts, a pair of electrooculographic (EOG) electrodes placed below the left eye and on the frontal processes of the left zygomatic bone were applied. Head movements were monitored continuously during the recordings using 5 head‐position‐indicator (HPI) coils.

Prior to the MEG recording, anatomical landmarks (nasion and left and right preauricular points), HPI coils and EEG electrode positions, and 100 (+/−5) additional scalp‐surface points were digitized using the Isotrak 3D digitizer (Polhemus Navigational Sciences, Colchester, VT). To ensure roughly equal distances between the scalp and the frontal and occipital sensors, a special cushion was used whenever necessary.

The stimuli were shown on a semi‐transparent back‐projection screen by a projector located outside the shielded room. The distance between a participant's eyes and the screen was 1.25 m.

### 2.8 Preprocessing

For the MEG signal, external magnetic interference was suppressed and head movements compensated for using the signal‐space separation (SSS) method implemented in the MaxFilter software (version 2.2; Elekta Oy, Helsinki, Finland) (Taulu & Simola, 2006). Thereafter, cardiac and ocular artifacts were projected out from the EEG and MEG data using the FastICA algorithm as implemented in the MNE‐Python software (Gramfort et al., 2013). Components corresponding to cardiac and ocular artifacts were excluded based on the visual inspection of their topographies and time courses. Epochs containing signal amplitudes greater than 12,000 fT. for magnetometers, 4,000 fT/cm for gradiometers and 150 μV for EEG electrodes were removed from the analysis automatically. The data were filtered to 0.5–20 Hz using a Hamming‐windowed finite impulse response filter whose length corresponded to 6.6 times the reciprocal of the shortest transition band and resampled at 125 Hz to minimize the number of features. To account for possible classification bias, the number of positive and negative outcome trials was equalized within each condition by excluding epochs of the class with higher trial count so that the time intervals between remaining trials were minimal. Trial counts for the within‐ and across‐condition classification analysis are presented in Supporting Information Table I.

### 2.9 Extracting epochs and reference events

The data were split into epochs of −200–600 ms for the evoked‐response analysis and 0–500 ms for time‐resolved classification and across‐condition generalization analysis. For each condition, the epochs were extracted relative to the corresponding reference event: in the *motor* condition at the onset of the button press; in the *bci* condition at the onset of the arrow indicating the BCI selection; and in the *feedback:expectancy* and *feedback:valence* conditions at the onset of the feedback cue.

In the analysis of the *feedback:valence* and *feedback:expectancy* conditions, only trials following correct decoding of the subject's intention by the BCI were used. *Feedback* trials that followed erroneous BCI performance were excluded from the analysis.

### 2.10 Probing the expectancy component of FRN

To assess the contribution of the *expectancy*‐specific component of FRN to the across‐condition generalization, we split the negative feedback trials into two categories based on the expectancy of the outcome. We defined negative feedback as *high surprise* whenever it resulted from picking the better alternative; conversely, negative feedback to choosing the lower‐value alternative was considered *low surprise*. It was much harder for the subjects to learn the preferences in trials containing two moderately valuable stimuli (66% vs. 33%) and two least valuable stimuli (33% vs. 20%) until very late in the experiment. Thus, in such trials the expectation to get positive feedback was relatively low as compared to high‐surprise negative feedback trials (e.g., when the most valuable stimulus [80%] resulted in negative feedback).

The behavioral results suggested that by the fourth experimental block (see Section 3.1) most of the subjects already have developed reasonable expectations about the value of the stimuli. Based on that we hypothesized that by experimental blocks 4 and 5 subjects had strong expectations to receive positive feedback after choosing the higher‐value stimuli. Thus, the rare negative feedback resulting from the probabilistic nature of these stimuli should be less expected (more surprising) compared to negative feedback resulting from the selection of less‐valuable stimuli for which such expectations were weaker. Thus, a classifier trained to discriminate such *high* versus *low surprise negative feedback* should capture the activity specific to the expectancy of an outcome, but not its valence. This analysis was performed only on negative feedback trials due to the low number of trials where positive feedback resulted from picking the low‐value stimuli. Trial counts for this analysis are summarized in Supporting Information Table I.

### 2.11 Probing the valence component of the FRN

To probe the *valence‐*specific component of FRN, we trained a classifier on a subset of positive and negative feedback trials grouped in such a way that the number of *high‐* and *low‐surprise* events was equal within each group. Thus, we ensured that the expectancy‐specific effect is diffused across positive and negative outcomes and the classifier only captures valence‐specific information. The time‐resolved within‐condition classification and across‐condition generalization procedures for this analysis were identical to the ones described above. Trial counts for this analysis are summarized in Supporting Information Table I.

### 2.12 Analysis of the evoked responses

**Figure 2:**
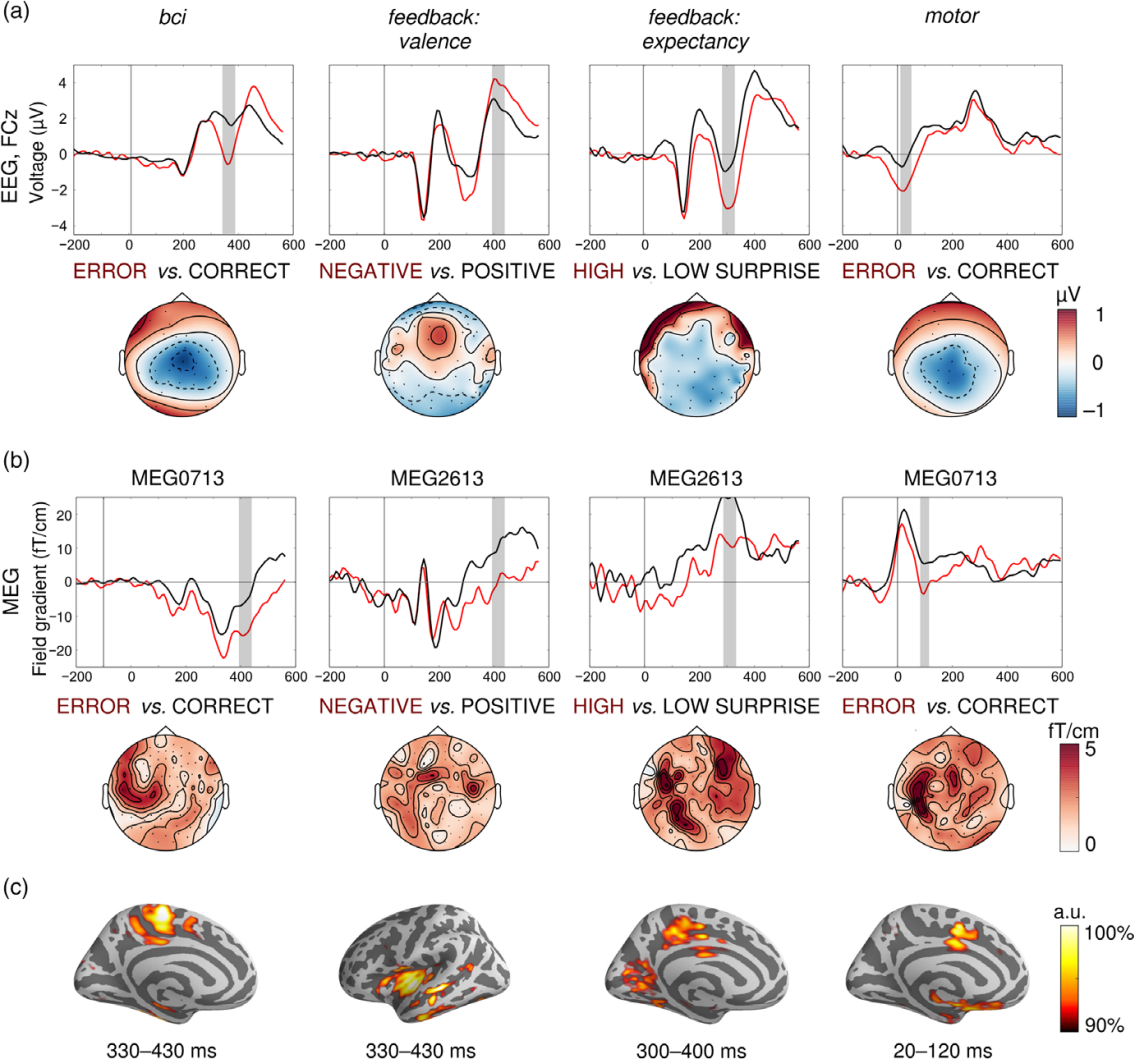
Grand‐averaged evoked responses to negative (red) versus positive (black) outcomes in bci (left, *N* = 14), feedback (middle, *N* = 14) and motor (right, *N* = 13) conditions. (a) EEG event‐related potentials at the FCz electrode with significant differences shaded in gray and the topographic maps representing the difference (negative vs. positive outcome) averaged across the time windows indicated by the gray shading. (b) MEG event‐related fields at sensors which display visible differences at latencies roughly corresponding to those of the EEG signal. MEG topographies represent the differences in the magnetic field gradients (norms of the planar gradiometer pairs) averaged across the time‐points indicated by the gray shading. (c) dSPM source estimates for grand‐average difference waves averaged over the 100‐ms time windows indicated below. Visualization threshold is 90% of the peak source activity [Color figure can be viewed at http://wileyonlinelibrary.com]

Evoked response analysis was performed to verify the presence of the evoked activity both in the EEG and MEG data. Evoked responses were baseline‐corrected by subtracting the mean amplitude value of the pre‐stimulus interval from the signal. In the EEG data, group‐level effects for all four conditions (*bci* performance*, feedback:valence, feedback:expectancy,* and *motor* selection) were probed using two‐tailed two‐sample permutation tests at the FCz electrode followed by maximum FWER correction for multiple comparisons. As no a priori hypothesis was available regarding the MEG sensor most sensitive to the magnetic counterpart of the ERN/FRN, no statistical analysis was performed on the evoked MEG data. For visualization purposes in Figure 2, we picked MEG sensors displaying the most prominent differences within each condition separately in the time windows corresponding to latencies observed in EEG.

### 2.13 Time‐resolved within‐condition classification

To identify time windows contributing to the discrimination within each condition (e.g., erroneous vs. correct *bci* performance; positive vs. negative feedback *valence*; high‐ vs. low‐surprise negative *feedback expectancy;* erroneous vs. correct *motor* selection), we trained a separate logistic regression classifier at each time point (0–500 ms with respect to the triggering event, sampled at 125 Hz) to discriminate between the respective outcomes and tested each of these classifiers on all other time points within this condition. This analysis was performed on the combined EEG–MEG sensor data for each subject separately. The resulting matrix contained within‐condition classification scores at each time‐point pair. These scores were obtained by computing the mean area under receiver‐operating characteristic curve (ROC AUC) via threefold cross validation. On each fold, a classifier was trained on 66% of the trials and tested on the held‐out 33%. Features from both the training and test sets were normalized by subtracting the mean and dividing by the standard deviation of the training set prior to the classification. The regularization parameter *C* for l2‐penalized logistic regression classifiers was set to 0.1 and no feature selection was performed.

### 2.14 Time‐resolved cross‐condition generalization analysis

To test for similarity between outcome‐specific evoked responses across *bci, feedback:valence, feedback:expectancy,* and *motor* conditions, time‐resolved cross‐condition generalization analysis was applied (Kaplan et al., 2015; King & Dehaene, 2014). At each time point (0–500 ms with respect to the triggering event, sampled at 125 Hz), a separate logistic‐regression classifier was trained to differentiate between negative and positive outcomes in one experimental condition (e.g., erroneous vs. correct *bci* performance) based on the single‐trial combined EEG–MEG sensor data for each subject separately. Then, the performance of each of these classifiers was tested at each time instance of the data measured at another condition to predict a different pair of class labels (e.g., erroneous vs. correct *motor* selection). The resulting matrix contained cross‐condition generalization scores at each time‐point pair for each subject separately. These scores were computed as the mean predictive ROC AUC across the six folds. On each fold, 66% of the trials from condition A were used to train the classifier. This classifier was then tested on all trials of condition B. The data from each condition were used as the training and test set alternatively (3 folds from A to B, and 3 folds from B to A). Similarly, to traditional cross‐validation, this sub‐sampling approach introduced a conservative bias onto the grand‐average ROC AUC estimates, reducing the variance of the classification score estimates and constraining the null‐distribution of ROC AUC estimates around the theoretical 50% chance level. Given the possible variability in evoked responses due to learning, this approach also allowed us to focus our analysis on the activity that was present in all trials.

Features from both the training and test sets were normalized by subtracting the mean and dividing by the standard deviation of the training set prior to the classification. The regularization parameter *C* for l2‐penalized logistic regression classifiers was set to 0.1 and no feature selection was performed.

### 2.15 Statistical analysis

The group‐level statistical significance of the within‐ and cross‐condition classification scores was assessed using one‐sample permutation test, followed by cluster correction for multiple comparisons (Maris & Oostenveld, 2007; Ojala & Garriga, 2009). The individual generalization score maps were transformed to deviations from the chance level by subtracting the theoretical chance level of 0.50 followed by a one‐sample one‐tailed permutation *t*‐test where we permuted the signs of de‐meaned AUC scores.

We clustered time‐point pairs where grand‐averaged within‐ or across‐condition classification scores were greater than the chance level with *p* < .01. Cluster mass was defined as the sum of the signed *t*‐values of all time points within the cluster. The Cluster *p*‐values were computed as a probability to observe a cluster of larger positive mass over 10,000 random permutations.

### 2.16 Source analysis

Source analysis was performed using only MEG data. Neural sources contributing to the observed differences in the evoked responses were estimated by computing dynamic statistical parametric mapping (dSPM) maps (Dale et al., 2000) for sources constrained onto the individual cortical surfaces and with orientations perpendicular to the local cortical surface (loose orientation constraint value of 0.2) as implemented in the MNE‐Python software (Gramfort et al., 2013). To this end, structural MRIs of each subject (not available for one) were segmented for the cortical mantle and cranial volume using the FreeSurfer software (http://surfer.nmr.mgh.harvard.edu/). The resulting cortical meshes were down‐sampled to 8,196 vertices to form the source spaces. A single‐compartment boundary element model describing the shape of the cranium was used as the volume‐conductor model. Noise‐covariance matrices were estimated from the 200‐ms pre‐stimulus intervals. Signal‐to‐noise ratio was defined as the ratio between traces of the respective covariance matrices.

Sources contributing to the observed differences within each condition were estimated by averaging the evoked activity over a 100‐ms time window centered around the peak of the respective evoked response, for each condition separately (*motor*: 20–120 ms, *feedback:expectancy*: 300–400 ms, *feedback:valence*: 330–430 ms, *bci*: 330–430 ms, see Figure 2c).

Similar dSPM source estimation procedure was performed for activation patterns, corresponding to the weights of the logistic regression classifiers, trained within 16 ms (three adjacent timepoints sampled at 125 Hz) around the maxima of the individual generalization maps. This source estimation was done to identify sources contributing specifically to the observed generalization effects.

To derive the activation patterns that contributed to the successful transfer of the classifiers across the conditions, we identified for each condition and for each subject the time point where this transfer resulted in the highest generalization score. We then extracted the classifier weight vectors of that and the two adjacent time points (in total 16 ms of the downsampled data) and multiplied them by the signal covariance matrix computed for this condition (Haufe et al., 2014) from the original (non‐downsampled) data within the same 16‐ms window for each subject and each condition individually to maximize the number of samples. We then applied the dSPM inverse solution identical to one described above to the obtained activation patterns and averaged the source estimates for each pair of conditions. Visualization threshold for active sources was set to correspond to 90% of the global maximum value.

## 3 RESULTS

### 3.1 Behavioral results

Subjects were able to learn the stimulus values during the BCI task. During the last two blocks (4 and 5) of the BCI task, 11 out of 14 subjects chose the more valuable stimulus in more than 75% of the trials, indicating learning and therefore likely development of expectations with regard to their choices.

In the motor task, the subjects chose the higher‐valued alternative in 76.6 ± 7.6% (mean ± *SD*) of trials. After the measurements, they were asked to rank the stimuli according to their value on a visual analog scale. The mean correlation of the reported and true order was 0.80.

### 3.2 BCI control

All subjects were able to effectively perform the BCI selection task; they reported incorrect decoding in 18.6 ± 3.3% of the trials (360 trials per subject in total). Supporting Information Table II summarizes the overall BCI control accuracy.

### 3.3 Evoked responses

In the EEG data, the average evoked responses at the FCz electrode differed significantly between the outcomes in all conditions (Figure 2, Supporting Information Table III), replicating the results of numerous previous EEG studies. As the primary goal of this analysis was to provide a timing reference for the cross‐condition generalization analysis, we do not discuss these results further for the sake of conciseness.

### 3.4 Time‐resolved within‐condition classification

**Figure 3:**
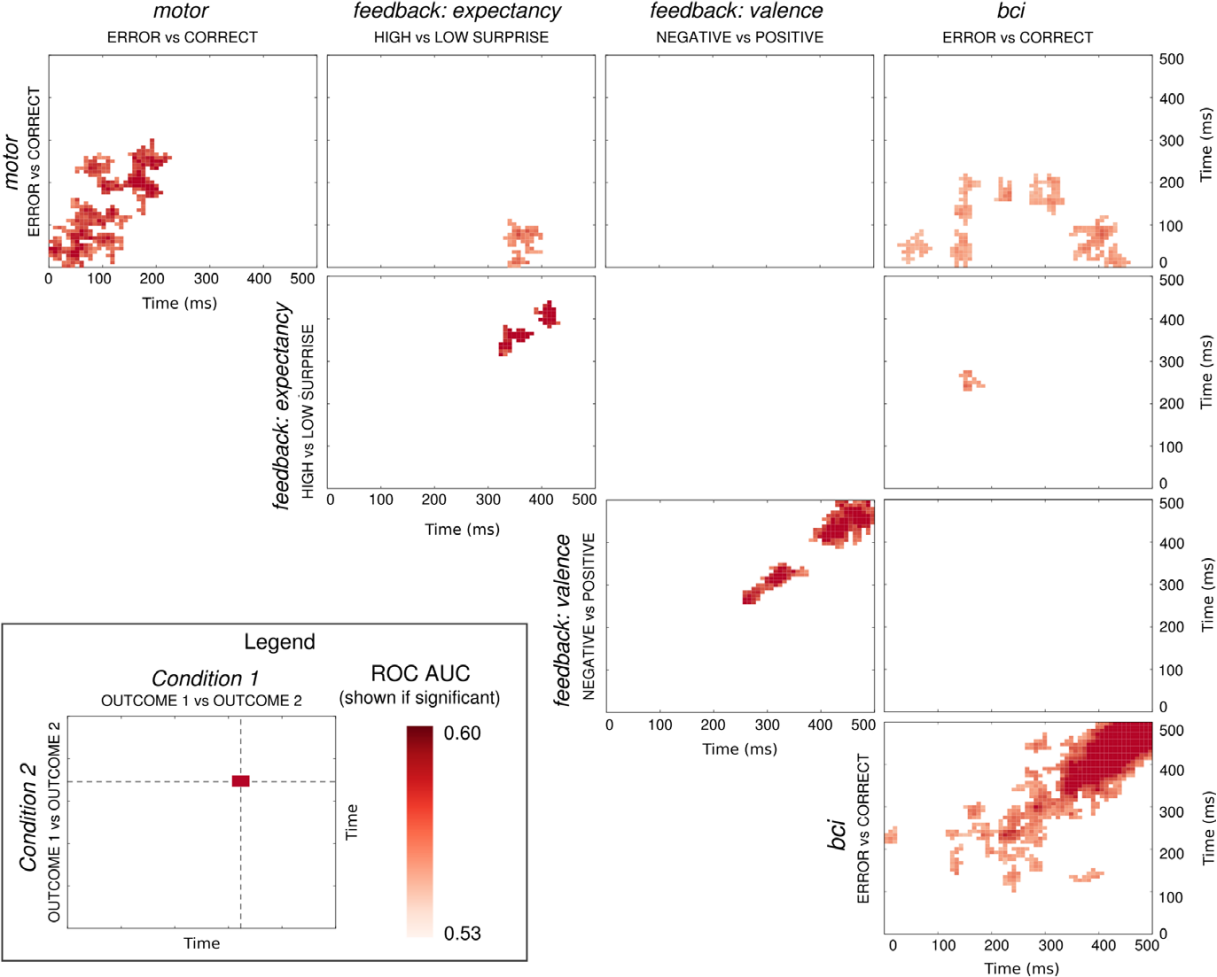
Time‐resolved within‐ and across‐condition generalization (combined EEG–MEG data). Colored areas represent significant clusters of within‐ (diagonal panes) and across‐ (off‐diagonal panes) condition ROC AUC scores. Cluster positions represent the time points in conditions indicated on the horizontal and vertical axes where the bi‐directional transfer of the classifier was significantly above chance. Generalization across *feedback*: *expectancy* and *feedback*: *valence* conditions could not be estimated reliably because these conditions could share data points. Non‐thresholded ROC AUC scores are presented in Supporting Information Figure 8 [Color figure can be viewed at http://wileyonlinelibrary.com]

To probe within condition classification (e.g., erroneous vs. correct *motor* selection), a separate classifier was trained on single trial EEG–MEG data at each time point (0–500 ms with respect to the triggering event) and tested on all other time points within the same condition. Group‐level permutation tests performed on individual within‐condition classification maps identified clusters of time points where the ROC AUC scores were significantly higher than the theoretical 50% chance level within all studied conditions (Figure 3; diagonal). Table 1 summarizes the results of within‐condition classification.

**Table 1:** Time‐resolved within‐condition classification (combined EEG–MEG)

Condition	*N*	Cluster size	Cluster mass, *t*	Cluster *p* value	Time‐window (ms)	Max grand‐average AUC score
*Bci*	14	18	67	.0496	176–216	65.1
		522	2,730	<.0001	192–496	
*Feedback:valence*	14	35	125	.0131	264–320	60.9
		247	1,024	.0002	336–496	
*Feedback:expectancy*	14	23	75	.0172	320–392	59.6
		42	140	.0042	264–352	
*Motor*	13	28	93	.0405	8–80	58.6
		244	843	.0055	96–360	

### 3.5 Time‐resolved across‐condition generalization

To test for similarity of evoked responses across the *motor*, *feedback:valence*, *feedback:expectancy*, and *bci* conditions, we trained a separate classifier to discriminate between outcomes on single trial EEG–MEG data at each time point (0–500 ms with respect to the triggering event) in one condition (e.g., erroneous vs. correct *motor* selection) and applied it to each time point of another condition (e.g., negative vs. positive *feedback*). Group‐level permutation tests performed on the individual cross‐condition generalization maps indicated multiple statistically significant clusters of ROC AUC scores when comparing *motor* versus *feedback:expectancy* and *motor* versus *bci* conditions (Figure 3; top row; Table 2). We also observed a significant generalization cluster when comparing *bci* versus *feedback:expectancy*. Table 2 summarizes the results of cross‐condition generalization analysis and individual maximum ROC AUC scores.

**Table 2:** Time‐resolved across‐condition generalization (combined EEG–MEG)

Condition	*N*	Cluster size	Cluster mass, *t*	Cluster *p* value	Time‐window (ms)	Time‐window (ms)	Max grand‐average AUC score
*Motor* vs. *feedback: expectancy*	13				*Motor*	*Feedback*	55.8
		68	219	.0003	8–112	304–376	
		15	46	.0489	312–352	352–392	
*Motor* vs. *feedback: valence*	13				*Motor*	*Feedback*	54.0
		n.s.	n.s.	n.s.	n.s.	n.s.	
*Motor* vs. *bci*	13				*Motor*	*BCI*	56.5
		39	129	.0068	8–96	8–128	
		31	108	.0099	8–96	96–128	
		18	69	.0283	112–176	104–128	
		25	90	.0151	136–192	184–216	
		19	60	.0375	152–184	232–296	
		19	58	.0407	8–64	272–312	
		109	377	.0003	8–112	304–424	
		31	114	.0092	136–184	304–352	
*Bci* vs. *feedback: expectancy*	14				*BCI*	*Feedback*	55.4
		12	41	.0468	112–128	208–248	
*Bci* vs. *feedback: valence*	14				*BCI*	*Feedback*	54.0
		n.s.	n.s.	n.s.	n.s.	n.s.	

This analysis was also performed using EEG and MEG data separately (Supporting Information Tables IV–V, Supporting Information Figure 7); the results were in line with those from combined EEG–MEG.

### 3.6 Source analysis

**Figure 4:**
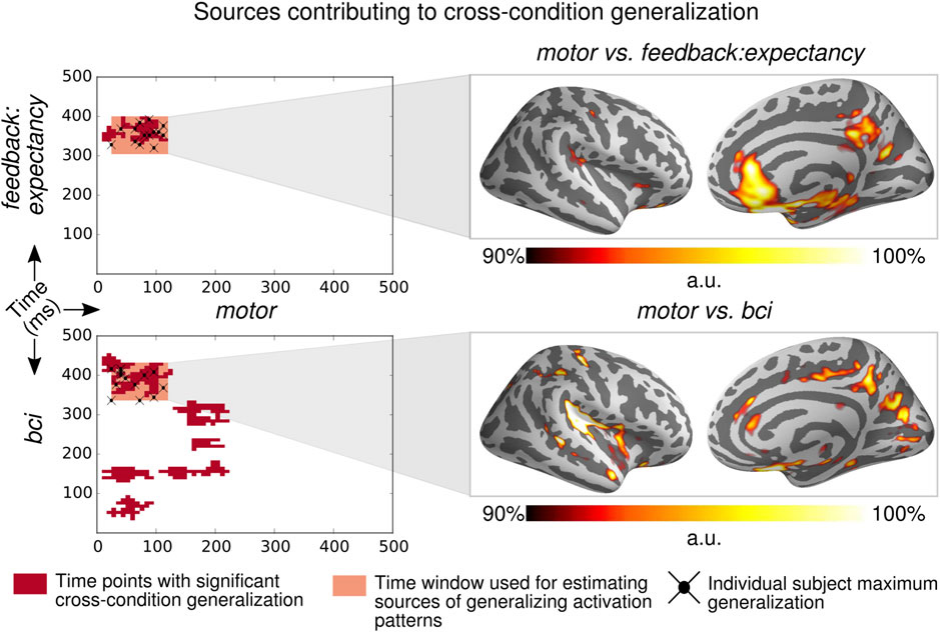
Estimation of neural sources informing the classifiers that generalize across *motor* versus *feedback:expectancy* (top) and *motor* versus *bci* (bottom) conditions. Red areas represent binary masks indicating significant clusters of ROC AUC scores. Black markers indicate time points where maximum generalization scores (searched within the shaded regions) were observed in each subject. Activation patterns were extracted using weights of logistic regression classifiers trained at these time points for each subject and condition separately. Source maps indicate grand‐averaged (normalized) source estimates of thus obtained activation patterns across subjects and conditions. Visualization threshold for source estimates is set to 90% of the peak activation value [Color figure can be viewed at http://wileyonlinelibrary.com]

Having identified the time points of maximum across‐condition generalization for each subject, we used the weights of the classifiers, trained at those time points, to extract patterns contributing to successful classification and estimated the neural sources producing these patterns (see Section 2 for details). Source analysis performed on the MEG data revealed that neural activity informing the successful classification originated from largely overlapping sources. In *motor* (20–120 ms) versus *feedback:expectancy* (300–400 ms) and *motor* (20–120 ms) versus *bci* (330–430 ms) conditions, we observed major contributions from bilateral dACC (BA 32), posterior cingulate cortex (PCC, BA 23,31) and right inferior temporal cortex including anterior insula (Figure 4). Results for clusters observed in different time windows in *motor* versus *bci* as well as in *bci* versus *feedback:expectancy* condition are presented in the Supporting Information (Supporting Information Figure 9–10).

## 4 DISCUSSION

The goal of this study was to probe whether the same neural process underlies the evaluation of action outcomes when operating a BCI, receiving feedback to a choice, or performing a motor task without feedback. To this end, we designed an experiment comprising a BCI‐controlled task (*bci*) involving learning from probabilistic *feedback*, followed by a separate *motor* task. We then tested the similarity of single‐trial event‐related responses to negative outcomes in EEG–MEG data.

We have observed that the classifiers trained to discriminate *erroneous* versus *correct motor* responses performed significantly above chance when predicting *erroneous* versus *correct BCI* performance and *unexpected* versus *expected* negative feedback from the EEG–MEG signal (Figure 3). The same was true when swapping the training and testing conditions.

The successful transfer of the classifiers between these conditions presents stronger quantitative evidence in favor of the similarity of the sources of the evoked responses to endogenous and exogenous errors, compared to the correlations between their spatial distributions alone. For such a transfer to work, two criteria need to be fulfilled simultaneously: spatial distributions of the underlying neural sources must be similar, and these sources must be functionally similar (discriminating action outcomes). Moreover, the fact that the latencies (typically 0–100 ms for ERN and 200–400 ms for FRN, see Figure 3) and the source locations (Figure 4) of these patterns, estimated from the data without any prior constraints, were in line with previous studies (for reviews see Walsh & Anderson, 2012; Weinberg et al., 2014) provides further support to the hypothesis of a general performance‐monitoring system in the brain.

Our results demonstrate that EEG–MEG signals following an erroneous *motor* response and the reception of highly surprising negative *feedback* are similar enough for a successful discrimination of analogous outcomes across these conditions. We trained separate classifiers to discriminate *feedback* trials according to the *expectancy* and the *valence* of the feedback *and* tested whether these classifiers could predict outcomes in *motor* or *bсi* conditions (see Section 2 for details). While both the *valence*‐ and *expectancy*‐based classifiers were able to discriminate outcomes within the respective conditions significantly above chance, only those classifiers that captured differences in brain responses to feedback *expectancy* generalized significantly to the *motor* condition. Our findings thus suggest that ERN and one component of FRN are generated by a common neural process that is sensitive to expectation violation but not to reward processing.

Extracting patterns of source activations associated with successful cross‐condition generalization revealed sources in the dACC (BA 32) and the PCC (BA 23,31), known to be involved in outcome processing (Agam et al., 2011; Becker, Nitsch, Miltner, & Straube, 2014; Keil et al., 2010; Miltner et al., 2003). In contrast to these studies, however, we specifically estimated sources that contributed to the transfer across the conditions (Figure 4).

Introducing a BCI in our study ensured that the cross‐condition generalization between *feedback* and *motor* trials is not confounded by the highly similar movement‐related activity that might otherwise result in spurious generalization. It also allowed us to study errors when operating a BCI, where the subject had only partial control over the task. Compared to passive viewing tasks used in previous studies (Martin & Potts, 2011)^,^ the BCI preserves the sense of agency (or control) that cannot be assured in a passive viewing paradigm. Thus, BCI errors reflect the loss of control rather than reward‐prediction error associated with classical FRN. This distinction was supported by the fact that no generalization between feedback valence and BCI trials was observed. However, the across‐condition generalization largely depends on the structure of noise covariance as well as that of signal. Thus, by the absence of across‐condition classification performance alone, we cannot exclude the possibility that the same neural sources are active and class‐discriminative.

We observed that a failure to control the BCI triggers essentially the same neural processes as committing errors in the motor task. Neural signals following the incorrect operation of a BCI system have been reported earlier, mainly in the context of using such responses as an additional control or learning signal for optimizing BCI performance (Buttfield, Ferrez, & Millán, 2006). A recent study demonstrated that neural sources contributing to brain responses to erroneous BCI performance indeed largely overlap with the putative sources of the FRN (Dyson, Thomas, Casini, & Burle, 2015). In addition, our cross‐condition generalization results indicate that these BCI*‐*related error responses and ERN share at least some of the underlying neural generators.

Previous studies have shown that the amplitudes of error‐ and feedback‐related evoked responses vary as learning progresses. FRN is most prominent early in learning as feedback is the most important source of task‐related information. Later, the response‐locked ERN becomes more prominent, indicating the development of subjects’ internal predictions regarding the trial outcome. Thus, it is not clear if ERN and FRN can occur within the same trials. To accommodate for this possibility, in our experimental design we ensured that (a) ERN and FRN were estimated in separate tasks; (b) the subjects had learned to prefer the more valuable stimuli in the BCI task before proceeding to the motor task (see Section 3.1); and (c) no feedback was presented during the motor task, ensuring that no further learning occurred. Thus, we argue that the observed ERN triggered by choosing the less valuable stimulus in the motor task was based solely on subjects’ preferences learned during the BCI task.

ERN‐like activity could occur also later in the BCI task on selection of the less valuable stimulus. But even in that case, the trial outcome could not be completely determined at the moment of target selection, because the feedback was delivered probabilistically.

Moreover, in the analysis of the feedback‐related activity, we only used trials where BCI decoded the subjects’ intentions correctly. By contrast, when comparing BCI‐correct versus BCI‐error responses we split the trials according to whether the system decoded subject's intentions accurately, and not based on whether or not the system chose the more valuable target.

Finally, training the classifiers on three random folds of each condition ensured that the classifiers captured activity that was present in all trials. Further studies should focus on the components of error‐ and feedback‐related activity that vary with learning. These design choices ensured that the successful transfer of the classifier between the domains was not confounded by the structure of the task.

Taken together, our findings indicate that motor errors, unexpected negative feedback and failures to control a BCI trigger the same neural process. This process is likely to detect a mismatch between an expectation and the actual outcome, and not the reward or punishment associated with these outcomes. Our results may thus provide an explanation to the observed discrepancies between ERN and FRN alterations in a number of neuropsychiatric populations. While expectancy‐specific processing is attenuated in schizophrenic patients, the reward‐specific component may still be intact, resulting in a different ERP shape (Morris et al., 2011).

Moreover, our results provide evidence that the same neural process is involved also when controlling a BCI, indicating that processing errors may be independent of the involvement of the motor system. Our findings thus provide grounds for using error‐related neural responses in optimizing BCI and neurofeedback protocols.

## 5 CONCLUSIONS

Our findings provide direct evidence of a shared neural system underlying the ERN and FRN responses. Moreover, our analysis indicates that the error‐related responses observed in a task void of motor output, that is, when using a BCI, still reflect activity in the same performance‐monitoring neural circuitry that is engaged for monitoring motor output. This shared activity is likely to be specific to expectation violation and not to reward processing.

## CONFLICT OF INTEREST

The authors declare no conflict of interest.

## Supporting information


**Supplementary Table I.** Trial count summary across conditions
**Supplementary Table II.** BCI control summary by subject.
**Supplementary Table III.** *Group‐level Event‐Related Potential analysis at FCz electrode*
**Supplementary Table IV.** Time‐resolved within‐condition classification (EEG and MEG)
**Supplementary Table V.** Time‐resolved across‐condition generalization (EEG and MEG)
**Supplementary Table VI.** Individual maximum scores in across‐condition generalization and the corresponding time points
**Supplementary Figure VII.** Time‐resolved within‐ and across‐condition generalization computed separately for EEG (top) and MEG (bottom) data. Colored areas represent binary masks indicating clusters of time‐points where the generalization within (diagonal) and across (off‐diagonal) the conditions indicated on the horizontal and vertical axes was statistically significant.
**Supplementary Figure VIII.** Time‐resolved within‐ and across‐condition generalization computed for combined EEG–MEG data. Colored areas represent ROC AUC scores estimating the generalization within (diagonal) and across (off‐diagonal) the conditions indicated on the horizontal and vertical axes.
**Supplementary Figure IX.** Estimation of neural sources informing the classifiers that generalize across *motor vs. bci* conditions. Black markers indicate time points where maximum generalization scores were observed in each subject within time‐windows indicated by shaded areas. Visualization threshold for source estimates is set to 90% of the peak activation value.
**Supplementary Figure X.** Estimation of neural sources informing the classifiers that generalize across *feedback:expectancy vs. bci* conditions. Black markers indicate time points where maximum generalization scores were observed in each subject within time‐windows indicated by shaded areas. In the bottom panel time‐window was identified based on results of generalization of the both studied condition to the *motor* condition. Visualization threshold for source estimates is set to 90% of the peak activation value.
