# Supplementary material for: Evidence for a general performance‐monitoring system in the human brain
Source: Hum Brain Mapp. 2018 Jul 4;39(11):4322–33. doi: 10.1002/hbm.24273 (PMC6220993; doi:10.1002/hbm.24273)
Supplement: Supplementary file 1 — Supplementary Table I. Trial count summary across conditions Supplementary Table II. BCI control summary by subject. Supplementary Table III. Group‐level Event‐Related Potential analysis at FCz electrode Supplementary Table IV. Time‐resolved within‐condition classification (EEG and MEG) Supplementary Table V. Time‐resolved across‐condition generalization (EEG and MEG) Supplementary Table VI. Individual maximum scores in across‐condition generalization and the corresponding time points Supplementary Figure VII. Time‐resolved within‐ and across‐condition generalization computed separately for EEG (top) and MEG (bottom) data. Colored areas represent binary masks indicating clusters of time‐points where the generalization within (diagonal) and across (off‐diagonal) the conditions indicated on the horizontal and vertical axes was statistically significant. Supplementary Figure VIII. Time‐resolved within‐ and across‐condition generalization computed for combined EEG–MEG data. Colored areas represent ROC AUC scores estimating the generalization within (diagonal) and across (off‐diagonal) the conditions indicated on the horizontal and vertical axes. Supplementary Figure IX. Estimation of neural sources informing the classifiers that generalize across motor vs. bci conditions. Black markers indicate time points where maximum generalization scores were observed in each subject within time‐windows indicated by shaded areas. Visualization threshold for source estimates is set to 90% of the peak activation value. Supplementary Figure X. Estimation of neural sources informing the classifiers that generalize across feedback:expectancy vs. bci conditions. Black markers indicate time points where maximum generalization scores were observed in each subject within time‐windows indicated by shaded areas. In the bottom panel time‐window was identified based on results of generalization of the both studied condition to the motor condition. Visualization threshold for source estimates is [file HBM-39-4322-s001.docx]

**Supplementary Material**

**Supplementary Tables**

**Supplementary table I.** Trial count summary across conditions

|  | **Feedback** | | | | | | **BCI** | | | **Motor** | | |
| --- | --- | --- | --- | --- | --- | --- | --- | --- | --- | --- | --- | --- |
|  | **Trials: Positive** | | **Trials: Negative** | | **Analysis** | | **Trials** | | **Analysis** | **Trials** | | **Analysis** |
| id | High surprise | Low surprise | High surprise | Low surprise | *expectancy* | *valence* | Error | Correct | *bci* | Error | Correct | *motor* |
| s01 | 32 | 112 | 46 | 81 | 46 | 128 | 54 | 267 | 108 | 47 | 272 | 94 |
| s02 | 15 | 140 | 50 | 52 | 32 | 60 | 58 | 249 | 116 | 46 | 139 | 98 |
| s03 | 11 | 95 | 37 | 26 | 50 | 44 | 31 | 166 | 62 | 50 | 97 | 79 |
| s04 | 42 | 123 | 49 | 67 | 54 | 168 | 57 | 281 | 114 | 36 | 118 | 72 |
| s05 | 12 | 180 | 51 | 46 | 42 | 48 | 52 | 284 | 104 | 40 | 199 | 80 |
| s06 | 11 | 161 | 59 | 42 | 38 | 44 | 40 | 274 | 80 | 48 | 156 | 96 |
| s07 | 24 | 154 | 53 | 56 | 36 | 96 | 64 | 272 | 128 | 6 | 109 | 12 |
| s08 | 42 | 153 | 52 | 57 | 42 | 168 | 36 | 294 | 72 | 76 | 148 | 152 |
| s09 | 9 | 161 | 54 | 50 | 42 | 36 | 70 | 268 | 140 | 78 | 134 | 124 |
| s10 | 4 | 65 | 19 | 18 | 30 | 16 | 27 | 101 | 54 | 32 | 172 | 64 |
| s11 | 31 | 110 | 48 | 79 | 48 | 124 | 52 | 268 | 104 | 46 | 148 | 92 |
| s12 | 26 | 176 | 53 | 42 | 34 | 104 | 38 | 277 | 76 | 41 | 169 | 82 |
| s13 | 30 | 134 | 40 | 63 | 42 | 120 | 41 | 255 | 82 | 40 | 152 | 80 |
| s14 | 23 | 153 | 55 | 68 | 44 | 92 | 57 | 298 | 114 | 62 | 162 | 124 |
| **mean** | **22.3** | **136.9** | **47.6** | **53.4** | **41.4** | **89.1** | **48.4** | **253.9** | **96.7** | **46.3** | **155.4** | **89.2** |

**Supplementary Table II**. BCI control summary by subject.

| **Subject** | **Introduced errors** | **Reported errors** | **Reported errors (%)** | **Actual errors (%)** |
| --- | --- | --- | --- | --- |
| s01 | 57 | 66 | 0.18 | 0.03 |
| s02 | 55 | 69 | 0.19 | 0.04 |
| s03 | 66 | 75 | 0.21 | 0.03 |
| s04 | 61 | 72 | 0.20 | 0.03 |
| s05 | 58 | 67 | 0.19 | 0.03 |
| s06 | 40 | 53 | 0.15 | 0.04 |
| s07 | 74 | 84 | 0.23 | 0.03 |
| s08 | 54 | 54 | 0.15 | 0.00 |
| s09 | 71 | 75 | 0.21 | 0.01 |
| s10 | 50 | 94 | 0.26 | 0.12 |
| s11 | 56 | 56 | 0.16 | 0.00 |
| s12 | 52 | 58 | 0.16 | 0.02 |
| s13 | 56 | 60 | 0.17 | 0.01 |
| s14 | 53 | 57 | 0.16 | 0.01 |
| **mean** | **57** | **67** | **0.19** | **0.03** |
| **std** | **9** | **12** | **0.03** | **0.03** |

**Supplementary Table III*.*** *Group-level Event-Related Potential analysis at FCz electrode*

|  | **Electrode** | **Time window (ms)** | **T-values, range** | ***p*-value, FWER** |
| --- | --- | --- | --- | --- |
| *bci* | FCz | 336–376 | –6.35 – –4.37 | 0.0375–0.0018 |
| *feedback: expectancy* | FCz | 316–340 | –4.94 – –4.16 | 0.0439–0.0099 |
| *feedback: valence* | FCz | 444–460 | 4.12 – 4.76 | 0.0439–0.0140 |
| *motor* | FCz | 32–56 | –3.79 – –3.90 | 0.0499–0.0399 |

**Supplementary Table IV.** Time-resolved within-condition classification (EEG and MEG)

| **EEG** | | | | | |
| --- | --- | --- | --- | --- | --- |
| **Condition** | **Cluster size** | **Cluster mass, t** | **Cluster *p*-value** | **Time-window (ms)** | **Max grand-average score** |
| *motor* | n.s. | n.s. | n.s. | n.s. | 56.7 |
| *feedback:valence* | 19 | 63 | 0.0423 | 400 – 456 | 58.7 |
| *feedback:expectancy* | 35 | 121 | 0.0205 | 264 – 352 | 60.4 |
| *bci* | 287 | 1143 | 0.0007 | 304 – 496 | 62.4 |
| **MEG** | | | | | |
| **Condition** | **Cluster size** | **Cluster mass, t** | **Cluster *p*-value** | **Time-window (ms)** | **Max grand-average score** |
| *motor* | 31 | 103 | 0.0376 | 8 – 64 | 59.5 |
|  | 402 | 1468 | 0.0006 | 40 – 304 |  |
|  | 42 | 139 | 0.0279 | 24 – 184 |  |
|  | 64 | 200 | 0.0195 | 200 – 419 |  |
| *feedback:valence* | 20 | 67 | 0.0452 | 280 – 320 | 60.5 |
|  | 210 | 833 | 0.0004 | 336 – 496 |  |
| *feedback:expectancy* | 16 | 55 | 0.0255 | 336 – 392 | 59.6 |
| *bci* | 26 | 110 | 0.0216 | 176 – 224 | 63.6 |
|  | 30 | 121 | 0.0195 | 240 – 304 |  |
|  | 477 | 2444 | 0.0003 | 256 - 496 |  |

**Supplementary Table V.** Time-resolved across-condition generalization (EEG and MEG)

| **EEG** | | | | | | | |
| --- | --- | --- | --- | --- | --- | --- | --- |
| **Condition** | **Cluster size** | **Cluster mass, t** | **Cluster *p*-value** | **Time-window (ms)** | **Time-window (ms)** | | **Max grand-average score** |
| *motor vs. feedback:expectancy* |  |  |  | *Motor* | | *Feedback* | 57.4 |
|  | 40 | 132 | 0.0045 | 8 – 80 | | 224 – 288 |  |
|  | 62 | 211 | 0.0016 | 8 – 80 | | 304 – 384 |  |
|  | 16 | 48 | 0.0449 | 16 – 48 | | 432 – 472 |  |
| *motor vs. feedback:valence* |  |  |  | *Motor* | | *Feedback* | 54.0 |
|  | n.s. | n.s. | n.s. | n.s. | | n.s. |  |
| *motor vs. bci* |  |  |  | *Motor* | | *BCI* | 56.8 |
|  | 24 | 76 | 0.0273 | 8 – 72 | | 64 – 104 |  |
|  | 49 | 179 | 0.0038 | 104 – 168 | | 104 – 184 |  |
|  | 20 | 64 | 0.0379 | 128 – 176 | | 264 – 296 |  |
|  | 24 | 77 | 0.0267 | 64 – 128 | | 272 – 304 |  |
|  | 205 | 773 | <0.0001 | 8 – 192 | | 280 – 416 |  |
| *bci vs. feedback:expectancy* |  |  |  | *BCI* | | *Feedback* | 57.6 |
|  | 76 | 266 | 0.0013 | 320 – 400 | | 296 – 408 |  |
|  | 39 | 133 | 0.0059 | 72 – 144 | | 312 – 384 |  |
|  | 38 | 130 | 0.0066 | 152 – 232 | | 312 – 376 |  |
|  | 19 | 62 | 0.0318 | 240 – 304 | | 384 – 400 |  |
|  | 18 | 58 | 0.0363 | 344 – 400 | | 424 – 448 |  |
|  | 30 | 100 | 0.0114 | 424 – 496 | | 432 – 464 |  |
| *bci vs. feedback:valence* |  |  |  | *BCI* | | *Feedback* | 55.3 |
|  | 19 | 64 | 0.0226 | 448 – 480 | | 416 – 472 |  |
| **MEG** | | | | | | | |
| **Condition** | **Cluster size** | **Cluster mass, t** | **Cluster *p*-value** | **Time-window (ms)** | | **Time-window (ms)** | **Max grand-average score** |
| *motor vs.*  *feedback:expectancy* |  |  |  | *Motor* | | *Feedback* |  |
|  | 57 | 204 | <0.0001 | 72 – 136 | | 288 – 384 | 55.8 |
|  | 17 | 52 | 0.0211 | 176 – 200 | | 328 – 384 |  |
|  | 14 | 45 | 0.0342 | 280 – 320 | | 328 – 368 |  |
|  | 17 | 53 | 0.0203 | 472 – 406 | | 336 – 384 |  |
|  | 18 | 66 | 0.0104 | 408 – 448 | | 344 – 392 |  |
|  | 24 | 81 | 0.0049 | 312 – 376 | | 352 – 302 |  |
| *motor vs.*  *feedback:valence* |  |  |  | *Motor* | | *Feedback* |  |
|  | n.s. | n.s. | n.s. | n.s. | | n.s. | 54.0 |
| *motor vs. bci* |  |  |  | *Motor* | | *BCI* |  |
|  | 16 | 50 | 0.0498 | 24 – 80 | | 8 – 32 | 54.9 |
|  | 20 | 69 | 0.0254 | 32 – 88 | | 96 – 128 |  |
|  | 18 | 68 | 0.0263 | 144 – 184 | | 184 – 208 |  |
|  | 21 | 74 | 0.0207 | 48 – 88 | | 240 – 272 |  |
|  | 18 | 56 | 0.0396 | 328 – 352 | | 240 – 296 |  |
|  | 21 | 69 | 0.0249 | 272 – 296 | | 256 – 304 |  |
|  | 23 | 76 | 0.0187 | 168 – 216 | | 264 – 304 |  |
|  | 36 | 125 | 0.0045 | 40 – 104 | | 328 – 376 |  |
| *bci vs.*  *feedback:expectancy* |  |  |  | *BCI* | | *Feedback* |  |
|  | n.s. | n.s. | n.s. | n.s. | | n.s. | 54.1 |
| *bci vs. feedback:valence* |  |  |  | *BCI* | | *Feedback* |  |
|  | n.s. | n.s. | n.s. | n.s. | | n.s. | 54.6 |
|  |  |  |  |  | |  |  |

**Supplementary Table VI.** Individual maximum scores in across-condition generalization and the corresponding time points

| subject | *motor vs. feedback:expectancy* | | | *motor vs. bci* | | | *bci vs. feedback:expectancy* | | |
| --- | --- | --- | --- | --- | --- | --- | --- | --- | --- |
|  | Time (ms) | | ROC AUC | Time (ms) | | ROC AUC | Time (ms) | | ROC AUC |
|  | motor | feedback: expectancy |  | motor | bci |  | bci | feedback: expectancy |  |
| s01 | 40 | 368 | 0.67 | 80 | 400 | 0.75 | 392 | 352 | 0.68 |
| s02 | 24 | 328 | 0.72 | 40 | 416 | 0.65 | 104 | 352 | 0.70 |
| s03 | 96 | 320 | 0.70 | 64 | 376 | 0.63 | 344 | 248 | 0.69 |
| s04 | 72 | 328 | 0.70 | 48 | 392 | 0.62 | 392 | 360 | 0.64 |
| s05 | 64 | 368 | 0.72 | 24 | 336 | 0.67 | 368 | 376 | 0.75 |
| s06 | 88 | 352 | 0.74 | 96 | 344 | 0.68 | 136 | 336 | 0.74 |
| s07 | 112 | 352 | 0.63 | 112 | 368 | 0.67 | 320 | 240 | 0.68 |
| s08 | 64 | 336 | 0.69 | 40 | 408 | 0.64 | 336 | 288 | 0.71 |
| s09 | 72 | 384 | 0.76 | 96 | 408 | 0.69 | 224 | 208 | 0.68 |
| s10 | 104 | 360 | 0.70 | 72 | 336 | 0.65 | 232 | 312 | 0.70 |
| s11 | 88 | 392 | 0.74 | 40 | 400 | 0.64 | 152 | 392 | 0.74 |
| s12 | 80 | 352 | 0.68 | 32 | 376 | 0.66 | 240 | 208 | 0.67 |
| s13 | 96 | 360 | 0.69 | 24 | 416 | 0.65 | 344 | 344 | 0.68 |
| s14 | 104 | 288 | 0.75 | 56 | 208 | 0.69 | 176 | 232 | 0.68 |
| mean | 78 | 349 | 0.71 | 59 | 382 | 0.66 | 268 | 303 | 0.70 |

**Supplementary figures**

**Supplementary Figure VII.** Time-resolved within- and across-condition generalization computed separately for EEG (top) and MEG (bottom) data. Colored areas represent binary masks indicating clusters of time-points where the generalization within (diagonal) and across (off-diagonal) the conditions indicated on the horizontal and vertical axes was statistically significant.


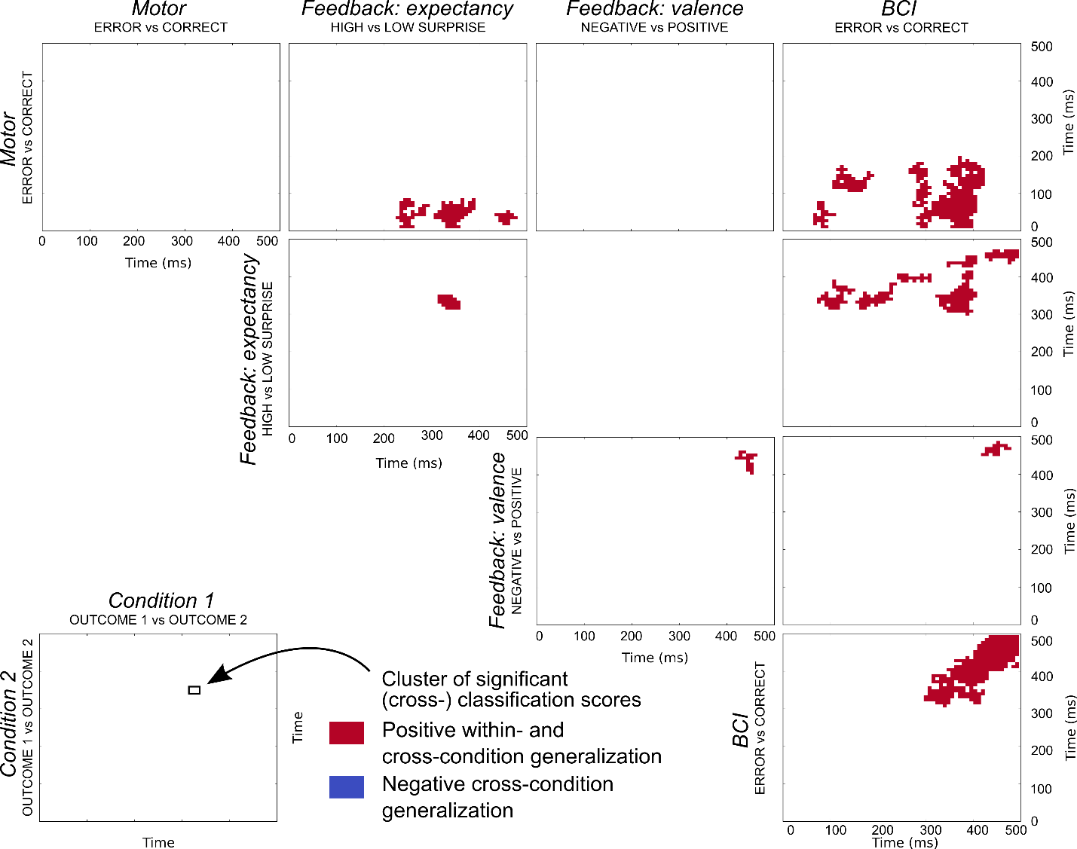

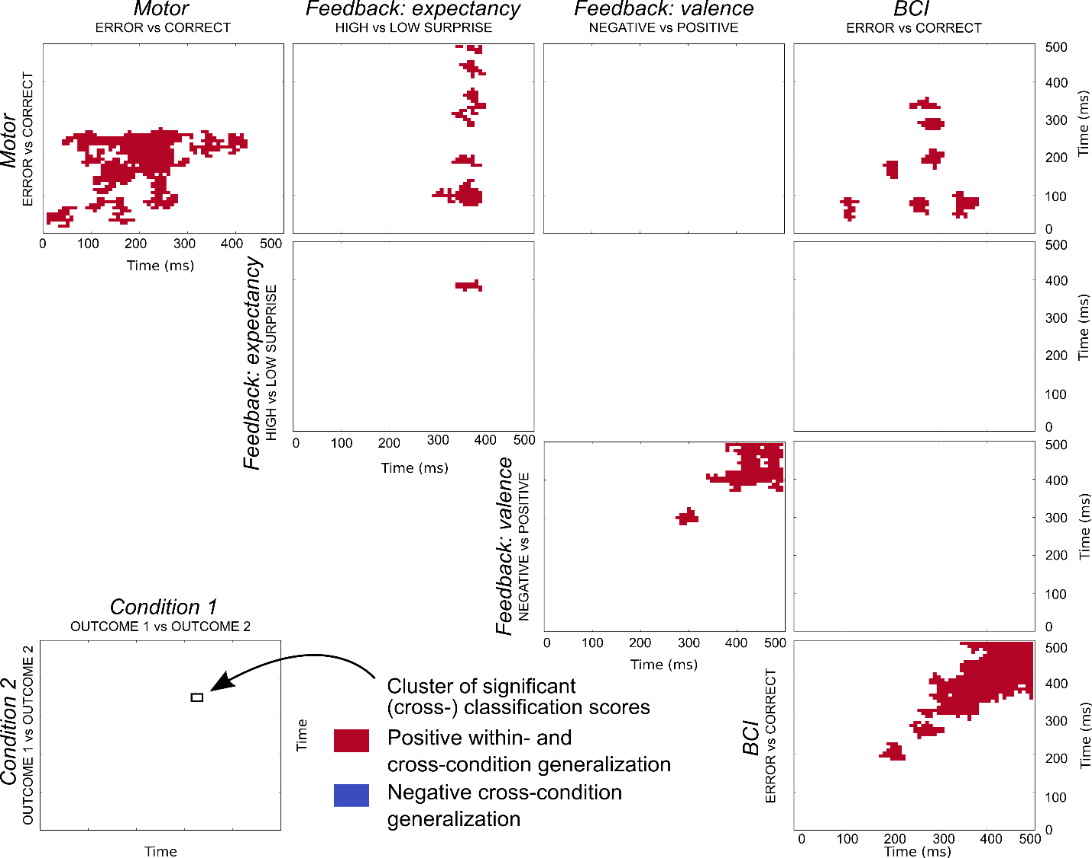


MEG only

EEG only

*
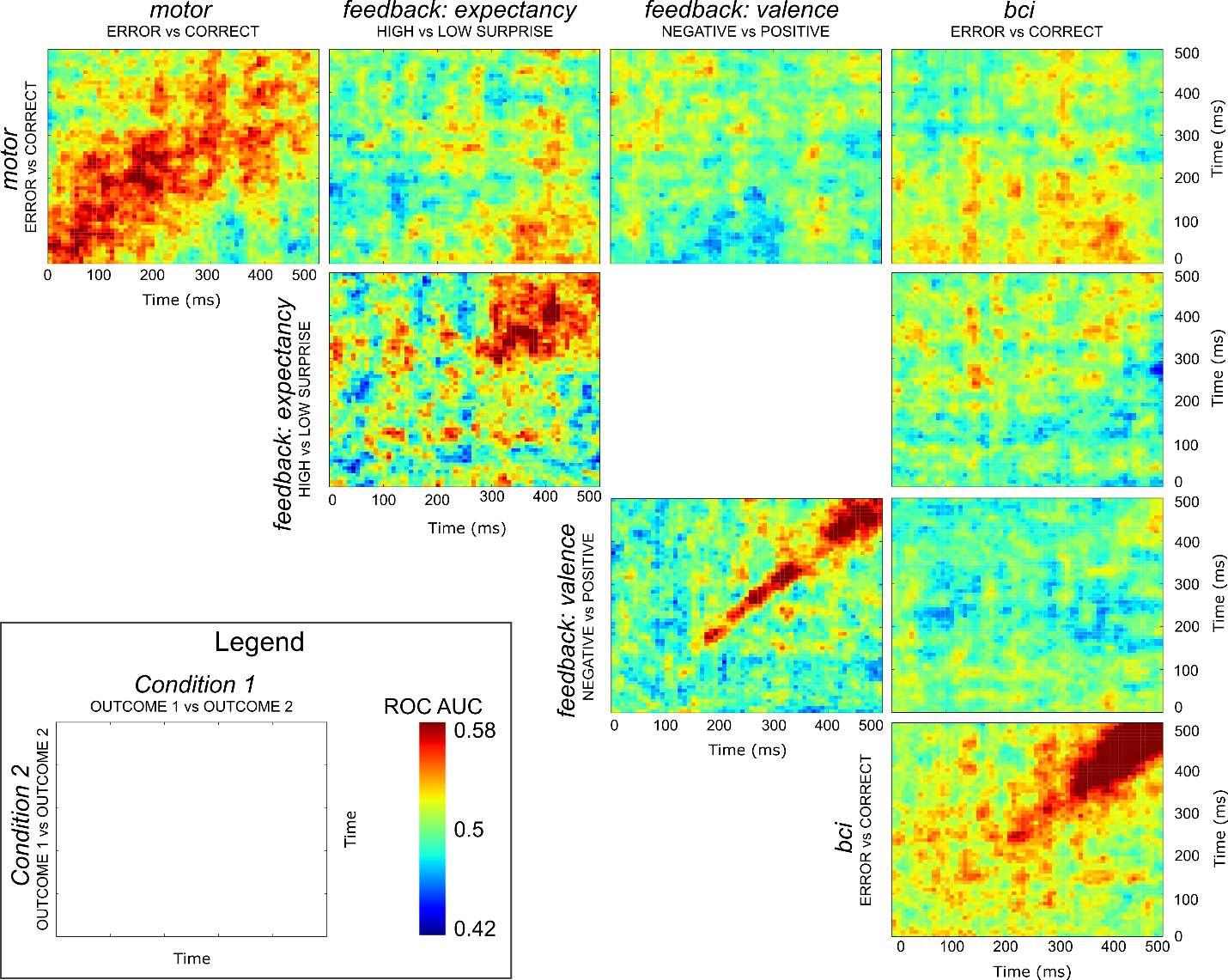
*

**Supplementary Figure VIII.** Time-resolved within- and across-condition generalization computed for combined EEG–MEG data. Colored areas represent ROC AUC scores estimating the generalization within (diagonal) and across (off-diagonal) the conditions indicated on the horizontal and vertical axes.

*
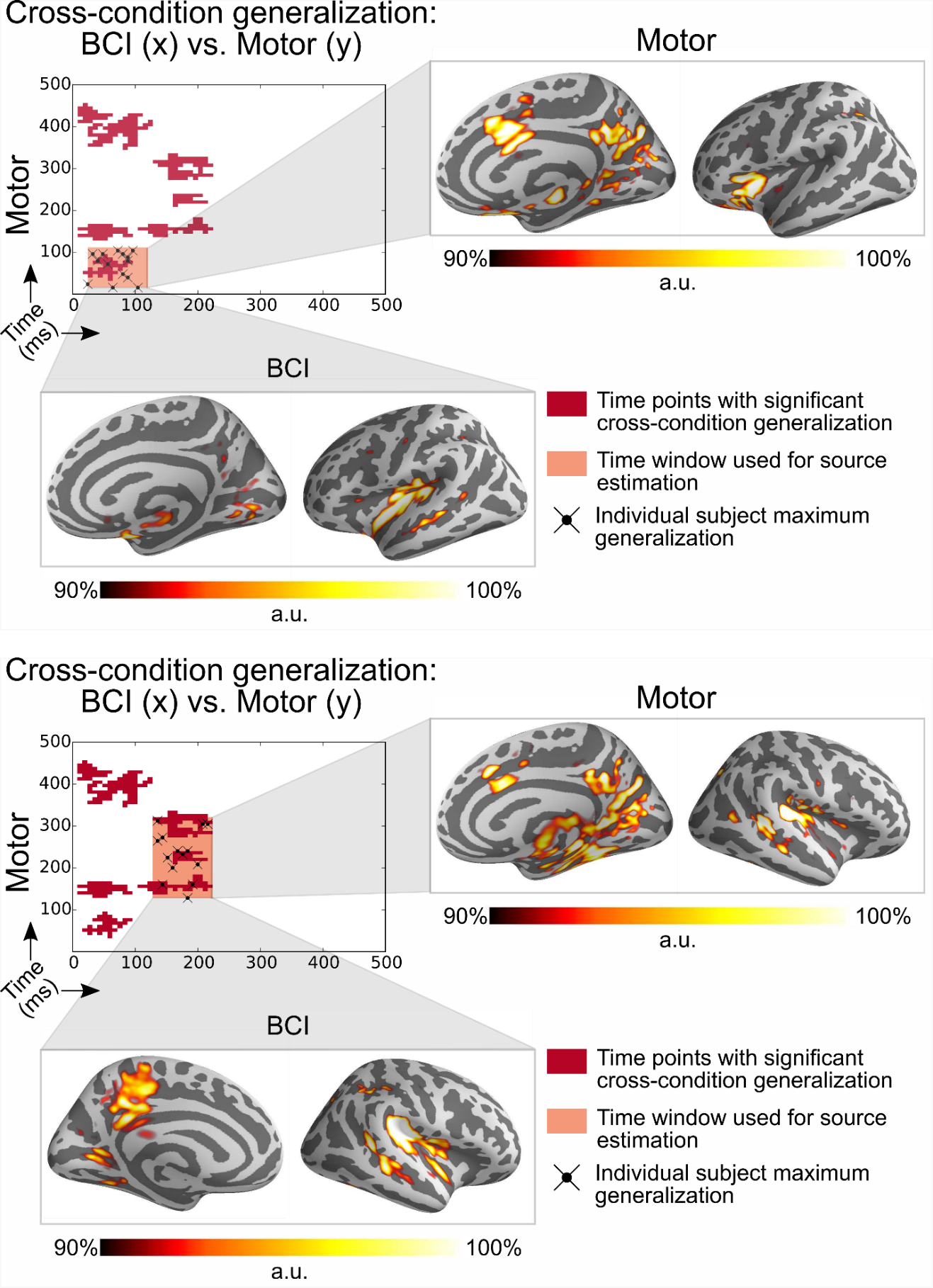
*

**Supplementary Figure IX.** Estimation of neural sources informing the classifiers that generalize across *motor vs.* *bci* conditions. Black markers indicate time points where maximum generalization scores were observed in each subject within time-windows indicated by shaded areas. Visualization threshold for source estimates is set to 90% of the peak activation value.

*
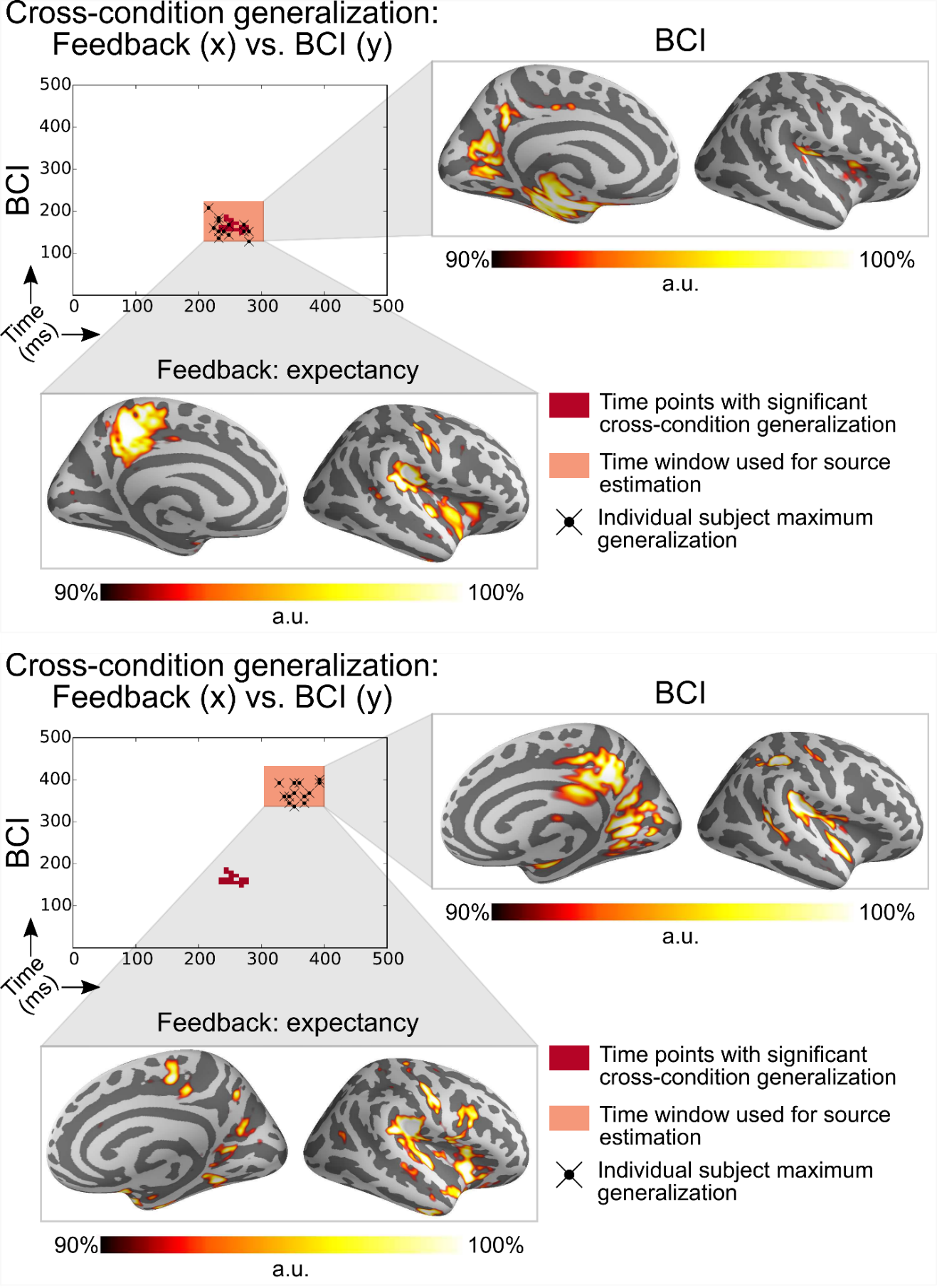
*

**Supplementary Figure X*.*** Estimation of neural sources informing the classifiers that generalize across *feedback:expectancy vs.* *bci* conditions. Black markers indicate time points where maximum generalization scores were observed in each subject within time-windows indicated by shaded areas. In the bottom panel time-window was identified based on results of generalization of the both studied condition to the *motor* condition. Visualization threshold for source estimates is set to 90% of the peak activation value.
